# Hybrid Visible Light and Ultrasound-Based Sensor for Distance Estimation

**DOI:** 10.3390/s17020330

**Published:** 2017-02-10

**Authors:** Jose Rabadan, Victor Guerra, Rafael Rodríguez, Julio Rufo, Martin Luna-Rivera, Rafael Perez-Jimenez

**Affiliations:** 1 IDeTIC, Universidad de Las Palmas de Gran Canaria, PCT Tafira, 35017 Las Palmas, Spain; vguerra@idetic.eu (V.G.); rrodrihe@gmail.com (R.R.); jrufo@idetic.eu (J.R.); rafael.perez@ulpgc.es (R.P.-J.); 2 Facultad de Ciencias, Universidad Autónoma de San Luis Potosi, Avda Salvador Nava S/N, 78290 San Luis Potosi, Mexico; mlr@uaslp.mx

**Keywords:** visible light communications, optical wireless communications, visible light positioning, cricket sensor, distance measurement

## Abstract

Distance estimation plays an important role in location-based services, which has become very popular in recent years. In this paper, a new short range cricket sensor-based approach is proposed for indoor location applications. This solution uses Time Difference of Arrival (TDoA) between an optical and an ultrasound signal which are transmitted simultaneously, to estimate the distance from the base station to the mobile receiver. The measurement of the TDoA at the mobile receiver endpoint is proportional to the distance. The use of optical and ultrasound signals instead of the conventional radio wave signal makes the proposed approach suitable for environments with high levels of electromagnetic interference or where the propagation of radio frequencies is entirely restricted. Furthermore, unlike classical cricket systems, a double-way measurement procedure is introduced, allowing both the base station and mobile node to perform distance estimation simultaneously.

## 1. Introduction

Indoor distance measurement has traditionally been a challenging problem for many indoor applications that require positioning information. Current location-based services demand highly accurate positioning systems, but electromagnetic Radio-Frequency (RF) propagation is severely affected in indoor environments, causing significant errors and poor resolution in systems based on Ultra-Wide Band (UWB) or Received Signal Strength Indicator (RSSI) [1,2,3]. Other short distance ranging solutions are based on the IEEE 802.15.4 standard using phase detection techniques [4], or estimating the time of arrival [5], unless it is severely affected by reflections or the presence of obstacles. Visible Light Communications (VLC) based positioning techniques, also known as Visible Light Positioning or VLP, are being proposed for these kinds of applications [6,7,8,9]. VLP provides an adaptive data transmission grid for cost-efficient guiding that could be used not only for visually impaired people in indoor scenarios [6], but also by different applications in many other scenarios. One example could be robot guidance inside an industrial facility (that can be heavily affected by electromagnetic (EM) noise) or positioning inside a building when security forces are under a bomb threat and jamming devices are activated, in order to avoid remote control or cell phone detonators. It takes advantage of the relative high switching speed of Light-Emitting Diode (LED) lamps, as well as other characteristics: low cost, high speed propagation signal, robustness against electromagnetic interference (intentional or not) or spectrum saturation, etc. As a commercial example, Phillips has recently presented, in collaboration with Carrefour [10], a beaconing system based on illumination lamps and a cell phone-based Optical Camera Communication receiver for guidance in a commercial center.

In this paper, a VLC distance-measurement scheme, based on Time-Difference of Arrival (TdoA) Cricket techniques [11,12,13,14] is introduced. The advantage of a hybrid optical and ultrasound measurement system relies on easier distance estimation, as processing is performed over a slower wave (acoustical signal), rather than a pure VLP system that requires high sampling speed and expensive data acquisition devices. In [15], authors also made use of both systems, but they performed distance estimation separately. The use of an optical signal instead of RF is suggested to avoid the aforementioned radio signal problems. Additional benefits include its application in some specific environments, such as underwater scenarios where RF becomes impractical (they suffer from high and prohibitive attenuation). Furthermore, the transmission and reception stages have been modified in order to provide distance measurement capabilities to both devices involved in the process.

In standard Cricket systems, only the receiver node devices are able to calculate the distance from the signal emitted by the transmitter node. The proposed system consists of the following nodes:

- Base station or transmitter node: is the reference block, with a fixed known position, from where the distance value will be estimated. Furthermore, it starts the measurement process emitting both optical and ultrasound signals, used by the mobile node for distance estimation.
- Mobile or receiver node: represents the other endpoint of the line to be measured, it calculates the distance from the signals generated by the base station. Additionally, it returns a new optical signal to the base station so as to also perform its own distance estimation.

Finally, as in other solutions, for a full distance measurement system, a trilateration structure [16] for positioning will be needed. It requires at least three base stations and one mobile node. The mobile node position can be obtained by means of a trilateration positioning calculation, using the information of the base stations’ positions and the estimated distances among the base stations and the mobile node. In order to assure the correct transmission of each base station without interference among them, a Time-Division Multiple Access (TDMA) scheme can be scheduled. As the time intervals between transmissions are fixed and known, the distance estimation algorithm can be easily modified to include these intervals in the calculations.

This paper is organized as follows. A description of the proposed method for obtaining the distance is presented in Section 2. Then a proof of concept demonstration is provided in Section 3 with an implemented prototype, while Section 4 provides a detailed description of the measurements and results obtained. Finally, a discussion about the given results and some conclusions are included.

## 2. System Description

**Figure 1:**
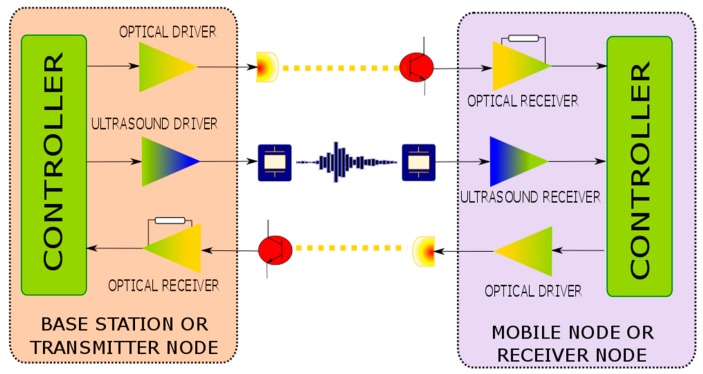
System block diagram.

The basic scheme of the proposed distance measurement protocol is presented in Figure 1. It can be observed that the transmitter node requires ultrasound and optical emitters, but also an optical detector, whilst the receiver node is composed of an ultrasound detector, an optical detector, and an optical emitter. Thus, the proposed scheme enables a one-way ultrasound link and two optical links. Without loss of generality, we consider the use of VLC technology for the optical links, as we intend to reuse the illumination fixtures as base stations of indoor positioning systems, however, we highlight that this scheme can also be implemented with other optical wavelengths, such as near infrared (NIR).

**Figure 2:**
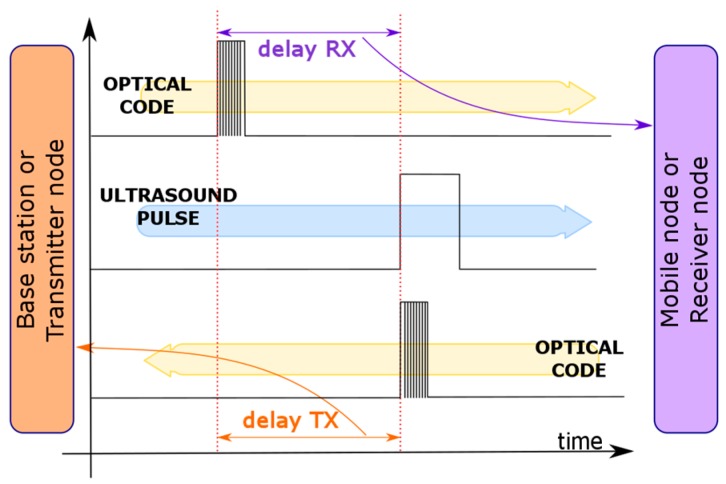
System operational chronogram.

In the measurement process, the base station starts sending simultaneously an optical code (e.g., an EUI-64 ID code) and an ultrasound pulse. Both transmissions are supposed to be simultaneous, but due to different delays, produced by the microcontroller instruction cycles and the electronic components used in each transmitter, the optical and ultrasound pulses are transmitted with a small delay among them. Unless this delay introduces an additional error in the measurement process, it can be easily calculated and its effect neglected. The mobile node receives the optical code and waits for the ultrasound signal reception. As the time for light propagation can be neglected, at least, when compared with sound propagation, it can be considered that the light is transmitted with a delay equal to zero. Since the relative delay between both signals in reaching the receiver node depends only on the ultrasound propagation, we can consider the distance from the base station to mobile node as proportional to that delay. Therefore, the estimated distance can be calculated using Equation (1):

(1) $$D=\frac{\Delta t}{\frac{1}{v_{S}}-\frac{1}{c}\;}\approx v_{S}\cdot \Delta t$$

where *D* defines the distance, Δt is the delay time, *c* is the speed of light and *v_S_* is the speed of sound that depends on the temperature. When the ultrasound signal is detected at the mobile node, it sends back an optical signal (again, it could be an ID code, but only the first bit is considered for time calculation), indicating that the ultrasound signal has reached the mobile node. Assuming that the optical propagation delay can also be neglected from the mobile node to the base station, the base station will also be able to estimate the distance by measuring the time difference between the instant of transmitting the ultrasound pulse (at the base station) and the arrival time of the optical signal sent from the mobile node (which is triggered by the transmitted ultrasound signal), providing a dual-side distance estimation capability. Figure 2 presents the corresponding chronogram depicting how the same delay values are obtained in the mobile node and in the base station.

### Mathematical Analysis

Based on the diagram presented in Figure 2, the time difference Δt between the optical and ultrasound signals can be estimated using the following equation. $t_{opt}$ and $t_{us}$ are the arrival times of the optical and acoustic signals, respectively.

(2) $$\Delta t=\left|t_{opt}-t_{us}\right|$$

Considering the time reference as the beginning of the optical emission, as well as the transmission chains of both the optical and ultrasound subsystems, the received signals before the detection stage can be expressed as Equation (3):

(3) $$V_{opt}=V_{tx}^{opt}\left(t\right)\times h_{tx}^{opt}\;\left(t\right)\times h_{ch}^{opt}\left(t\right)\times h_{rx}^{opt}\left(t\right)+n^{opt}\left(t\right)\;V_{us}=V_{tx}^{us}\left(t-t_{\mu C}\right)\times h_{tx}^{us}\;\left(t\right)\times h_{ch}^{us}\left(t\right)\times h_{rx}^{us}\left(t\right)+n^{us}\left(t\right)$$

where $V_{tx}^{opt}\left(t\right)$ and $V_{tx}^{us}\left(t\right)$ are the optical and acoustic excitation signals. The optical signal would typically be a pulsed signal, whilst the acoustic signal would be sine-like. $h_{tx}^{opt}\;\left(t\right)$ and $h_{tx}^{us}\;\left(t\right)$ are the optical and acoustic transmitters’ impulse responses, respectively. This response comprises both the amplification chain and transduction. In white LED lamps, phosphor or perovskite would introduce a certain persistence. Regarding acoustic transducers, ultrasonic emitters behave as bandpass filters. The VLC indoor impulse response (even in Line-of sight or LOS scenarios, it presents tails due to multiple reflections that extend tens of nanoseconds [17]) is taken into account in $h_{ch}^{opt}\left(t\right)$. On the other hand, in ultrasound links, due to the nature of pressure waves, the maximum theoretical bandwidth is just a few kHz [18] ($h_{ch}^{us}\left(t\right))$, limiting the ranging rate of a positioning system based on the proposed device; Priyantha’s cricket system presents the same limitation. $t_{\mu C}$ is the delay between the emitted signals, and depends on the microcontroller clock. $h_{rx}^{opt}\;\left(t\right)$ and $h_{rx}^{us}\;\left(t\right)$ are the optical and acoustic receiver impulse responses, respectively. Finally, $n^{opt}\left(t\right)$ and $n^{us}\left(t\right)$ are additive white Gaussian noise or sources.

In the case of the optical channel, all the involved signals are low pass filtered, introducing a slight delay on the received signal when enough power is emitted. On the other hand, both ultrasound emitter and receiver are bandpass filters due to their piezoelectric response (usually very narrow). As can be inferred from the receiver chain diagram, the detection is sensitive to the voltage threshold $V_{th}^{i}$ used and to the receiver’s noise power. Mathematically it can be modelled as Equation (4):

(4) $$t_{opt}=\inf\arg\left\{V_{opt}\left(t\right)\geq V_{th}^{opt}\right\}\;t_{us}=\inf\arg\left\{V_{us}\left(t\right)\geq V_{th}^{us}\right\}$$

**Figure 3:**
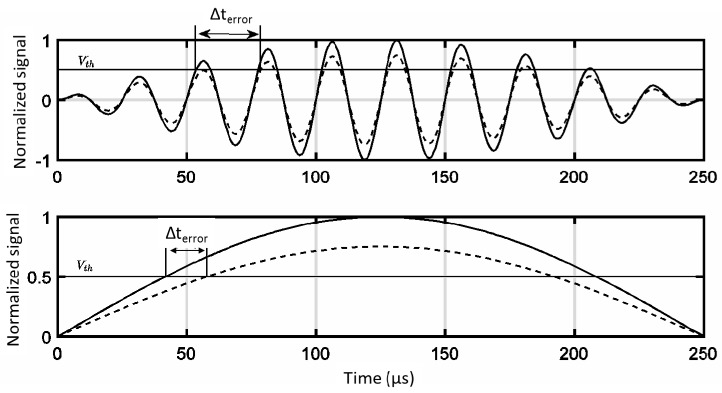
Effect of the received power on the estimation of the travelling time for the ultrasound signal. We have considered two cases: using a hard detector (**upper**) and an envelope-based detector (**lower**).

Taking into account the shape of the received signal, the delay errors on the detection stage could be neglected on the optical signal when compared to the same effect on the acoustic subsystem. In this case, the received signal would be sine-like and the delay errors would be proportional to the period of the transmitted signal. Furthermore, these delay errors would be distance-dependent and each cycle implies an 8 mm error on distance estimation (at 343 m/s of sound speed), complicating the achievement of a sub-centimeter system without a statistical post-processing adjustment stage. Figure 3 illustrates this dependency.

## 3. System Implementation

**Figure 4:**
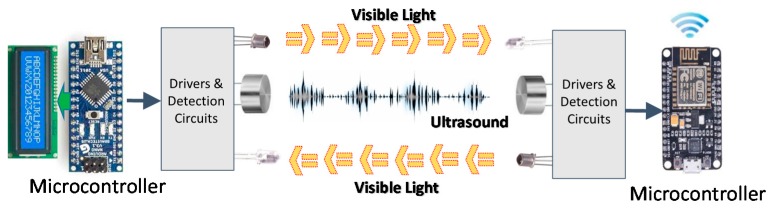
Operational scheme of the proposed system.

As a proof of concept, a prototype based on the proposed distance measurement scheme has been implemented. The aim of this prototype is to validate the procedure, not to present a fully reliable system. In this way, basic circuits and components have been used, which fulfill the prototype requirements. The real-time measurements and distance calculation are performed on microcontroller units (MCU), one on each side of the system. We have used an Atmel ATMega328P (Arduino Nano, Torino, Italy) for the base station and a NodeMCU (based on the ESP8266 chip, Guangzhou, China) for the mobile node. Moreover, the built system includes a liquid crystal display (LCD) and a Universal Serial Bus (USB) connection at the base station, in order to present the measured results. A wireless network connection has also been included in the mobile node for presenting the measured results. Figure 4 shows the scheme of the implemented system.

As it was previously stated, in the transmitter node a time counter starts when the light pulse and ultrasound signal are simultaneously sent. Since a MCU is used, an almost negligible delay is introduced between both pulses, as they are generated sequentially by the MCU and some instruction cycle delay is introduced. However, this delay is fixed by the MCU high frequency and accurate system clock, so we can consider it as a constant. Therefore, this delay can be easily considered at the receiver side calculation and does not introduce errors in the distance estimation. When the optical signal—only a pulse for this proof of concept—is detected at the mobile node, it starts its own time counter (it can be assumed that because of the speed of light, the time counters in both sides of the system start at almost the same time). As the ultrasound wave is slower than the light wave, the mobile node stops its time counter when the ultrasound signal is detected. Then, an optical pulse is immediately sent back to the transmitter node. The distance estimation in the mobile node is performed using the measured delay between the optical and ultrasound pulses. Furthermore, as the sound speed is highly affected by temperature, a sensor is included in the mobile node (in this case, an AOSONG DHT22 (Guangzhou, China)), in order to estimate the local sound speed and to compensate the propagation-speed error. The correction follows that shown in Equation (5):

(5) $$V_{S}=331.5+0.60714T\;\left(m/s\right)$$

where T is the temperature in Celsius degrees. When the base station node receives the optical signal sent by the mobile node, it stops its time counter and calculates its relative range. Therefore, both devices should obtain the same delay for calculating the distance with a minimum error coming from the delay of the optical propagation. Additionally, for the MCUs, each station has a hybrid (optical and ultrasound) communication layer. The ultrasound transducer is a Daventech 400ST/R160 (Tweedale Court Industrial Estate, Madeley, Telford, UK) with a 40 kHz clock signal generated by the microcontroller and a MAX232 integrated circuit-based driver. The ultrasonic receiver is based on the same transducer as the emitter, with a signal amplifier (Sony CX20106A, Tokyo, Japan), connected to the NodeMCU. This amplifier generates one pulse for each received ultrasound train of pulses, and a demodulation process of the 40 kHz carrier amplitude modulated (AM) ultrasound signal is then performed.

**Figure 5:**
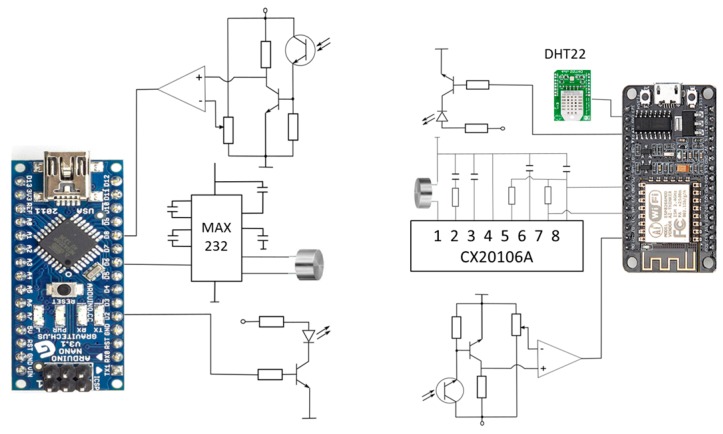
Electronic diagram of the implemented system, base (**left**) and remote (**right**) stations.

The optical link is based on commercial white LEDs, with a BPW40 phototransistor (Telefunken, Frankfurt am Main, Germany) and a comparison block for pulse shaping as the receiver. For practical systems, more complex communication schemes have to be introduced. Higher ranges require more powerful emitter stages and higher sensitivity receivers. Additionally, strategies for the reduction of the ambient light effects have to be provided, such as new modulation schemes or sectored receivers. If the range of the system needs to be increased, it would require the incorporation of higher power drivers at the transmitters and higher gain schemes at the receiver. Figure 5 represents the basic scheme of the whole implemented prototype.

## 4. Results

**Figure 6:**
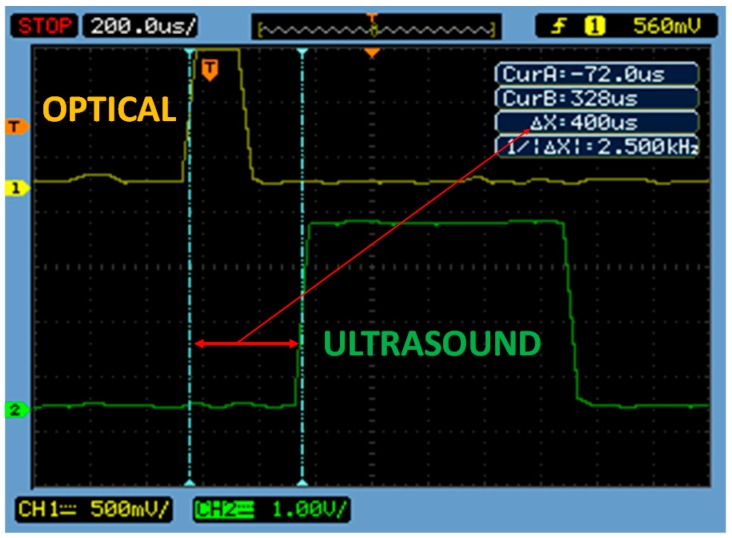
Experimental delay measurement, received by the mobile node (note that the scope denotes us for µs).

**Figure 7:**
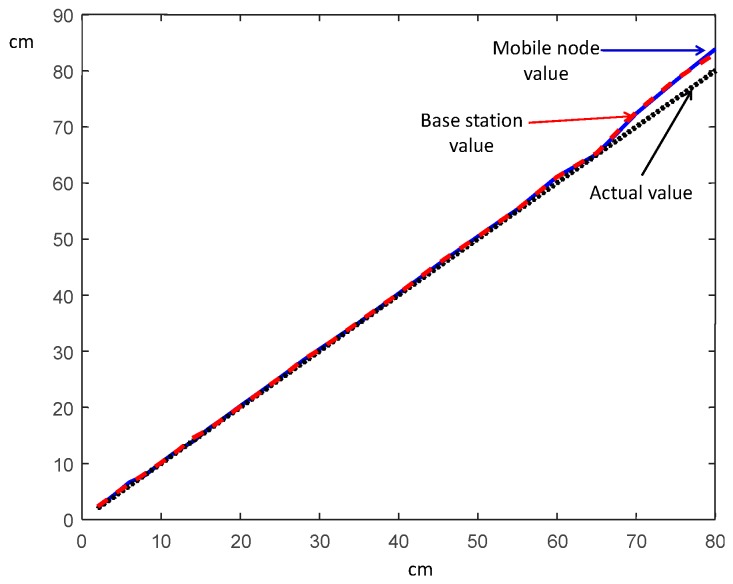
Measured versus real distance, at both the base and the remote stations.

This section presents the experimental results of the implemented system. The test setup is similar to that shown in Figure 4, where the measurement devices are aligned and the mobile node changes its position along a line. Figure 6 shows an experimental delay measurement, where the optical and ultrasound pulses, received by the mobile node, are illustrated. A delay between the two signals of Δ*X* = 400 µs is observed. Introducing this value of Δt in Equation (1) along with the sound speed (0.0343 cm/s) gives an estimated distance of 13.72 cm; for this evaluation the distance between terminals was set to 14 cm. Different distances up to 80 cm were measured whose results, obtained in both base and mobile stations, are presented in Figure 7. It can be observed that the relative error value remains below 2%, except at the operational limit of this system (as it was a proof of concept with low-gain configuration, it was set to 80 cm), where the error grows to 5%. Error is also significant for ultra-short distances (below 5 cm), where the relative error grows due to the minimum resolution limits of this implementation (measured values rose up to 17% at 3 cm).

## 5. Discussion

There are four main challenges usually kept in mind when dealing with indoor positioning systems:

- Accuracy (depending on the application requirements): the harshness of indoor environments on signal propagation, (caused by obstacles, wandering people, shadowing), makes it hard to achieve accuracy. Eventually, it will also be necessary in some study cases to provide not only position in a coordinate system, but also orientation.
- Scalability: Indoor environments often contain a large number of physical objects and a large density of people, all requiring a location. Hence, an indoor location system needs to scale well with the number and the density of users of the system. This is especially true for large scenarios such as airports or dense commercial areas.
- User privacy: The ability to obtain user location without tracking previous positions is important for preserving user privacy.
- Ease of deployment: The location system should be easy to deploy, configure, and maintain. The amount of manual configuration and precise placement should be as small as possible, while accuracy considerations have been discussed in the results section. Ease of maintenance also implies low power consumption (when it is powered by batteries).

We can now discuss the evaluation of the proposed system based on this metric. Privacy is guaranteed by the inherent security capabilities of the VLC system. It offers additional security when compared to radio frequency systems due to the nature of the light signals that, in contrast to RF signals, cannot be interfered with, nor read through walls. We can even imagine a scenario in which someone tries to produce an intentional error in a distance measurement by using a fake beacon signal from an external radio emitter. Regarding ease of deployment, a pre-installed facility can be used as the illumination network. The key factor is that the delay measurement is performed over the delay of the ultrasound signal, so less complex sampling of the received signal is required, compared to trying to evaluate the delay of an optical transmission, and lower cost devices can be used. Furthermore, the necessity of maintaining synchronization among the lamps is avoided. Scalability is the main problem for these devices, but can be achieved when different users receive the optical code from a base station, all of them will be waiting to “hear” the ultrasound ping and calculate the delay to locate themselves independently, waiting for the signals from the lamps.

## 6. Conclusions

This work has presented a new distance measurement scheme based on a TDoA technique that uses a dual optical-ultrasound system. This proposed system uses the same principle as the cricket technology but introduces some modifications, which provide advantages for some specific application scenarios. In contrast to traditional cricket systems, the proposed scheme measures the distance at the mobile node but also at the base station node, providing distance measurement capability to both devices involved in the process. It also introduces reinforced security compared with traditional RF systems, as each room can be considered as an isolated cell, as light does not propagate through walls. It also can be employed in scenarios where RF systems are not practical, such as underwater systems. As a proof of concept, we have implemented a basic TDoA visible light ultrasound sensor prototype in order to validate the proposed distance measurement scheme. Results show that both base station and mobile node devices are able to calculate the distance between them, with similar accuracy (about 2% error). These results can be easily improved (mainly in terms of range and accuracy) by introducing more powerful and efficient circuits and components. Future work will develop a complete positioning system and study the effects of the channel and interference effects on the accuracy and performance of the system. The main application area of this technique is for short-range distance estimation for location-based services, even in environments where RF communications are restricted, such as in water.
